# Lead-Free (1 – *x*)Ba(Zr_0.2_Ti_0.8_)O_3_ – *x*(Ba_0.7_Ca_0.3_)TiO_3_‑Based Ferroelectrics with Diffuse Phase Transitions for Sustainable Electrocaloric Applications

**DOI:** 10.1021/acs.jpcc.5c03396

**Published:** 2025-09-11

**Authors:** Krishnarjun Banerjee, Wanting Hu, Zixuan Wu, Xuyao Tang, Haixue Yan

**Affiliations:** 4617 School of Engineering and Materials Science, Queen Mary University of London, Mile End Road, London E1 4NS, U.K.

## Abstract

The electrocaloric effect of a ferroelectric material is an effective technique for solid-state refrigeration. Although the peak value of the adiabatic temperature change (Δ*T*) due to the electrocaloric effect in normal ferroelectrics is found near the Curie point (*T*_c_), ferroelectrics with a diffuse phase transition are always desirable due to their wide temperature span of Δ*T*. In this work, the electrocaloric effect in sustainable Pb-free weak relaxor ferroelectrics, (1 – *x*)­Ba­(Zr_0.2_Ti_0.8_)­O_3_ – *x*(Ba_0.7_Ca_0.3_)­TiO_3_ (*x* = 0.19 and 0.21), with diffuse phase transition is reported. The diffuseness of each composition is determined based on the fitting of the temperature-dependent dielectric permittivity data to the Lorenz-type quadratic law. Interestingly, the maxima of Δ*T* of the compositions are observed at much higher temperatures than the temperature at maximum dielectric permittivity (*T*_m_) and freezing temperature (*T*_f_), which work as the effective *T*_c_ of a relaxor ferroelectric. The wide temperature span (40 K) and high Δ*T* (0.8 K) of the *x* = 0.19 composition show the potential of this material for solid-state refrigeration applications. Moreover, the effects of diffuse phase transition on Δ*T* and its temperature span are discussed in (1 – *x*)­Ba­(Zr_0.2_Ti_0.8_)­O_3_ – *x*(Ba_0.7_Ca_0.3_)­TiO_3_-based ferroelectrics, which will help to design Pb-free electrocaloric materials with a diffuse phase transition for a wide working temperature range.

## Graphical abstract

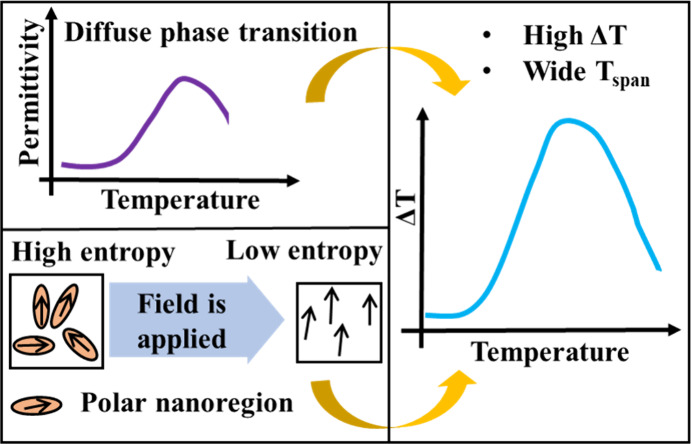

## Introduction

The reversible temperature change (Δ*T*) of a ferroelectric material during the application or removal of an electric field is known as the electrocaloric (EC) effect. The EC effect of ferroelectrics is becoming attractive due to its applications in solid-state refrigeration.^1^ The EC effect is useful for cooling applications in microelectronics, sensors, and medical specimen preservation.^2^ Most importantly, the hazardous gases that are used in traditional vapor compression refrigeration systems can be avoided in the EC-based solid-state refrigeration technique. The Δ*T* of a ferroelectric with the change in applied electric field under adiabatic conditions is directly related to the isothermal entropy change (Δ*S*).^1^ Various ferroelectrics like Pb­(Zr_1–*x*_Ti_*x*_)­O_3,_ ^3^ (1 – *x*)­Pb­(Zn_1/3_Nb_2/3_)­O_3_ – *x*PbTiO_3,_ ^4^ (1 – *x*)­Pb­(Mg_1/3_Nb_2/3_)­O_3_ – *x*PbTiO_3,_ ^5^ Pb­(Sc_1/2_Ta_1/2_)­O_3,_ ^6^ BaTiO_3_,^7^ 0.82­(Na_0.5_Bi_0.5_)­TiO_3_ – 0.18­(K_0.5_Bi_0.5_)­TiO_3_,^8^ Ba_0.85_Ca_0.15_Zr_0.10_Ti_0.90_O_3_,^9^ etc. are studied for solid-state cooling applications. However, the practical applications of EC materials face challenges such as low Δ*T* as well as concerns related to the high cost and/or toxicity of the constituent elements.

Usually, the displacive ferroelectric with a first-order phase transition shows the peak value of Δ*T* (Δ*T*_max_) just above or in the vicinity of the Curie point (*T*_c_), but such a type of phase transition restricts the working temperature range of the EC material. On the other hand, the relaxor ferroelectric with a diffuse-type phase transition is always desirable due to its broad transition around *T*_m_ (temperature at maximum permittivity), which helps to widen the temperature span (*T*_span_) of Δ*T* but limits its dielectric breakdown strength associated with the relaxor behavior. Therefore, research on the EC performances of the weak-relaxor ferroelectric with a diffuse-type phase transition is demanding.^10,11^ Among the lead-free ferroelectrics, the (1 – *x*)­Ba­(Zr_0.2_Ti_0.8_)­O_3_ – *x*(Ba_0.7_Ca_0.3_)­TiO_3_ (denoted as (1 – *x*)­BZT – *x*BCT)-based ceramics with a diffuse-type phase transition are gaining attention as promising EC materials.^12–14^ The diffuse-type phase transition in temperature-dependent dielectric data indicates the potential of these materials to produce a high Δ*T* over a broad *T*_span_.^15^ By definition, the temperature range where Δ*T* maintains the value equal to or greater than 80% of its maximum value is known as the *T*_span_.^16^ As the composition with a diffuse-type phase transition often suffers from low Δ*T*, it is challenging to attain high Δ*T* and wide *T*_span_ in a single composition. The EC performances of *x* = 0.3 and *x* = 0.7 in (1 – *x*)­BZT – *x*BCT can be exemplified in this case. For *x* = 0.3, a high Δ*T* of 0.3 K is observed in (1 – *x*)­BZT – *x*BCT in a wide temperature span, but the temperature span becomes narrow when Δ*T* slightly increases to 0.33 K for *x* = 0.7 composition.^15^

In this work, considering the effect of local inhomogeneity on the diffuse-type phase transition nature, we have designed the cation-site disorder (1 – *x*)­BZT – *x*BCT-based compositions to attain a broad *T*_span_. Simultaneously, the local compositional inhomogeneity creates polar nanoregions (PNRs). The high density of PNRs enables the high entropy state at zero electric field, which can change to the low entropy state under the presence of an electric field and produce a significant change in entropy, leading to high Δ*T*.^17^ Therefore, considering the combined influence of the diffuse phase transition on *T*_span_ and the dipolar contribution of PNRs to Δ*T*, in this study (1 – *x*)­BZT – *x*BCT where *x* = 0.19 and 0.21, ceramics (denoted as BCZT19 and BCZT21, respectively) are designed to attain high Δ*T* with broad *T*_span_. Simultaneously, we have discussed how a diffuse-type phase transition affects the position of Δ*T*_max_ and *T*_span_ in (1 – *x*)­BZT – *x*BCT-based ferroelectrics, which will help to design new EC materials with a wide working temperature range.

## Experimental Details

Ba_0.943_Ca_0.57_Zr_0.162_Ti_0.838_O_3_ (denoted as BCZT19) and Ba_0.937_Ca_0.063_Zr_0.158_Ti_0.842_O_3_ (denoted as BCZT21) ceramics were prepared through the solid-state method. The raw powders BaCO_3_ (Sigma-Aldrich, ≥99.9%), CaCO_3_ (Sigma-Aldrich, ≥99.9%), ZrO_2_ (Sigma-Aldrich, ≥99.5%), and TiO_2_ (Sigma-Aldrich, ≥99.8%) were dried at a temperature of 200 °C overnight. After that, the dried powders were weighed according to stoichiometric ratios and mixed using a ball mill. The slurries were dried and calcined at 800 °C for 3 h. The 5 wt % poly­(vinyl alcohol) was mixed with the calcined powder; after mixing, the powder was pressed into pellets and heated at 650 °C for 2 h to remove the binder. The green pellets were sintered at 1450 °C for 4 h. The density of all sintered pellets was tested by Archimedes’ principle in water. The relative density of the ceramics was higher than 96%. The room temperature crystal structure of the crushed samples was examined by an X-ray diffractometer (XRD, Xpert-Pro). The pellets were coated with Ag on both sides and fired. The dielectric permittivity and loss were measured as a function of temperature by using an LCR meter (Agilent, 4284A) attached to a furnace. A ferroelectric hysteresis measurement unit (NPL, UK) was used to examine the polarization-electric field loops at a frequency of 10 Hz and different temperatures. In order to measure the temperature properly, the ceramic samples were kept in a silicone oil bath during the measurement of the polarization-electric field loops. The temperature was measured by using a K-type thermocouple when the silicone oil temperature was stable and homogeneous. The heating rate was about 2 °C/min between the two measured temperatures, with a dwell time of 5 min maintained before we collected polarization-electric field data at each temperature. The accuracy of the temperature measurement is ± 0.1 °C. During the measurement of temperature-dependent dielectric permittivity, the heating rate of 1 °C/min was used to generate sufficient data points so that the temperature corresponding to the maximum permittivity can be measured accurately.

## Results and Discussion

Figure 1a displays the room temperature XRD patterns of the BCZT19 and BCZT21 compositions. The Bragg reflections are indexed according to the standard CaTiO_3_ perovskite structure (JCPDS 75-2100). The presence of any impurity phase was not detected in the XRD patterns.

**Figure 1:**
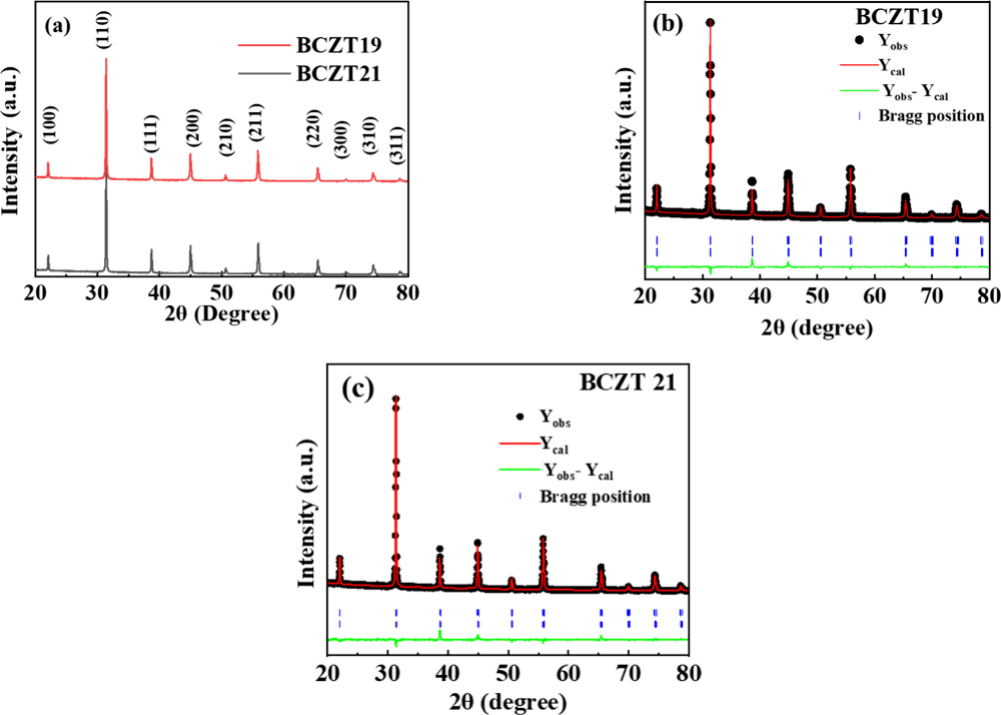
(a) Room temperature XRD patterns of BCZT19 and BCZT21 compositions. Refined XRD patterns of (b) BCZT19 and (c) BCZT21.

The *P*4*mm* (tetragonal) + *Amm*2 (orthorhombic) model was used to perform the XRD refinement. Rietveld refined XRD patterns of BCZT19 and BCZT21 are shown in Figure 1b,c, respectively. All of the XRD peaks of both compositions are fitted well with a double-phase model with a better goodness of fitting parameter (χ^2^). The structural parameters obtained after refinement are listed in Table 1.

**Table 1:** Phase Composition and Refined Lattice Parameters of the BCZT19 and BCZT21 Ceramics

compositions	lattice parameters (Å) for *P*4*mm* phase	lattice parameters (Å) for *Amm*2 phase	phase fraction and χ2
BCZT19	*a* = *b* = 4.0296 Å	*a* = 4.0308 Å	*P*4*mm* = 64.93%
	*c* = 4.0451 Å	*b* = 5.7045 Å	*Amm*2 = 35.07%
	α = β = γ = 90°	*c* = 5.7082 Å	χ^2^ = 3.20
		α = β = γ = 90°	*R*_P_ = 8.41, *R*_WP_ = 11.01
BCZT21	*a* = *b* = 4.0301 Å	*a* = 4.0294 Å	*P*4*mm* = 66.00%
	*c* = 4.0413 Å	*b* = 5.7010 Å	*Amm*2 = 34.00%
	α = β = γ = 90°	*c* = 5.7074 Å	χ^2^ = 3.27
		α = β = γ = 90°	*R*_P_ = 9.07, *R*_WP_ = 12.05

Figure 2a,b shows the temperature-dependent dielectric permittivity (ε′) and dielectric loss (tanδ) of BCZT19 and BCZT21 at various frequencies, respectively. As observed from the figure, the ε′ curves exhibit a broad transition around *T*_m_ (temperature at maximum ε′), which indicates the diffuse phase transition. The values of *T*_m_ of BCZT19 and BCZT21 are found to be 333 and 328 K at 1 kHz, respectively. It is possible to confirm the diffuse-type phase transition of the systems based on the modified Curie–Weiss law:^18^

$$\frac{1}{\varepsilon^{\prime }}-\frac{1}{\varepsilon_{m}^{\prime }}=\frac{(T-T_{m}{)}^{\gamma }}{C}$$ 1

where *C* is the Curie–Weiss constant, ε_m_^′^ is the maximum value of permittivity. Based on the value of γ, the diffuseness of a diffuse-type phase transition can be confirmed, i.e., ‘γ’ is 1 for normal ferroelectric and 2 for ferroelectric with diffuse-type phase transition. As the ‘γ’ parameter of the modified Curie–Weiss law distinguishes the normal ferroelectric transition and diffuse-type phase transition, the ‘γ’ values of the designed compositions are examined to confirm the diffuse-type phase transition. Figure 3a,b shows the plot of $\ln\left(\frac{1}{\varepsilon^{\prime }}-\frac{1}{\varepsilon_{m}^{\prime }}\right)$ vs ln­(*T* – *T*_m_) of BCZT19 and BCZT21, respectively. The values of ‘γ’ obtained after fitting for BCZT19 and BCZT21 at 1 kHz are 1.81 ± 0.00 and 1.75 ± 0.01, respectively. The γ value of BCZT19 is higher than BCZT21. Here, the concentration of BZT plays an important role in enhancing the diffuseness because BZT^19^ shows a higher value of ‘γ’ than BCT.^20^ The reason behind the broad transition around *T*_m_ is due to the distribution of the local Curie points of the microregions, which are created due to the local compositional inhomogeneity. The substitution of A-site Ca^2+^ and B-site Zr^4+^ at the cation sites of BaTiO_3_ is the reason for the diffuse-type phase transition in BCZT19 and BCZT21. The Curie points of the microregions follow a Gaussian-type distribution around a mean value.^21,22^

**Figure 2:**
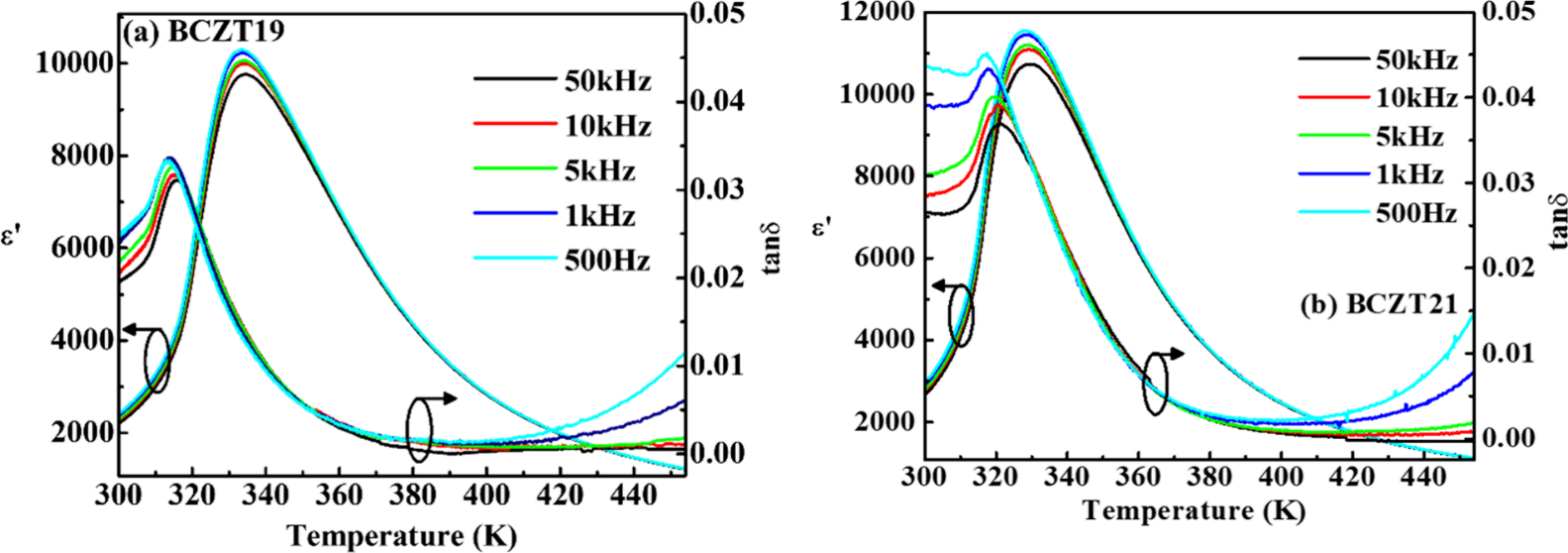
Temperature-dependent ε′ and tanδ of (a) BCZT19 and (b) BCZT21 at various frequencies.

**Figure 3:**
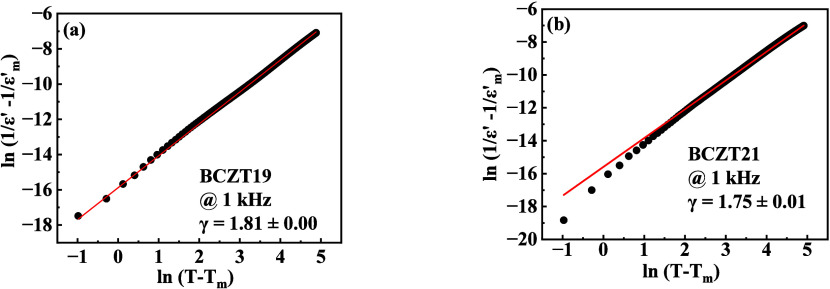
Plot of $\ln\left(\frac{1}{\varepsilon^{\prime }}-\frac{1}{\varepsilon_{m}^{\prime }}\right)$ vs ln­(*T* – *T*_m_) of (a) BCZT19 and (b) BCZT21. The solid red line denotes the fitting toeq [Disp-formula eq1]. The values of ‘γ’ are evaluated after fitting.

Bokov et al. proposed a quadratic formula to analyze the ferroelectric with diffuse-type phase transition.^22^ The formula is given below:

$$\frac{\varepsilon_{A}^{\prime }}{\varepsilon^{\prime }}=1+\frac{(T-T_{A}{)}^{2}}{2\delta^{2}}$$ 2

Here, *T*_A_, ε_A_^′^, and δ are the fitting parameters. For the particular values of ε_A_^′^ and *T*_A_, the ε′ – *T* data perfectly follow the abovementioned empirical relation.^23^ The ‘δ’ values indicate the broadness of the ε′ around *T*_m_ and confirm the diffuse-type phase transition of a composition.^23^ The frequency-independent characteristics of ‘δ’ obtained after fitting make this method more useful to determine the diffusiveness correctly. Figure 4a,b shows the ε′ – *T* curve and fitting to eq 2 for BCZT19 and BCZT21, respectively. The obtained values of ‘δ’ for BCZT19 and BCZT21 after fitting are 31.5 K ± 0.1 and 28.1 K ± 0.1, respectively.

**Figure 4:**
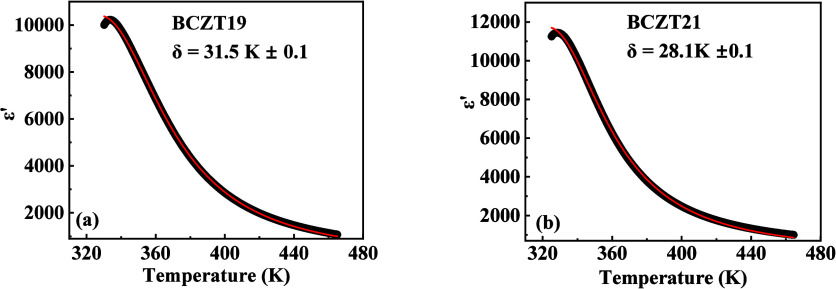
Dielectric permittivity vs temperature graphs of (a) BCZT19 and (b) BCZT21 at 1 kHz. The solid red curve denotes the fitting to eq 2.

Along with the diffuseness around *T*_m_, a little shifting of *T*_m_ toward the higher temperature with the increasing frequency is also noticed. The presence of the PNRs with different relaxation frequencies is responsible for the dispersive behavior of the temperature-dependent dielectric. The cation-site disorder in BCZT19 and BCZT21 creates chemical inhomogeneity at the local sites. The local compositional fluctuations are the source of the random local electric fields as proposed by Westphal, Kleemann, and Glinchuk.^24^ The local random electric field destroys the long-range domain order and leads to the dispersive nature of the dielectric permittivity.^25^ Brajesh et al. showed the presence of PNRs in BCZT-based materials based on the dispersive nature of the dielectric permittivity.^26^ In a relaxor ferroelectric, PNRs are created at the Burns temperature during cooling and fluctuate among the possible equivalent polarization directions. According to the dipolar glass model, with the decreasing temperature, the interactions between the PNRs increase and lead to the slowing down of the fluctuation, and at freezing temperature (*T*_f_), their dynamics become frozen.^27^ The *T*_f_ is calculated from the variation of *T*_m_ with frequency (*f*) according to the Vogel-Fulcher law:

$$f=f_{0}\exp\left(\frac{-E_{a}}{k_{B}(T_{m}-T_{f})}\right)$$ 3

where *f*_0_ is the pre-exponential factor, *E*_a_ is the activation energy, and *k*_B_ is the Boltzmann constant. Figure 5a,b shows the plot of ln­(*f*) vs *T*_m_ of the compositions. The calculated values of *T*_f_ of BCZT19 and BCZT21 are 331 and 322 K, respectively. During cooling, as the temperature approaches to *T*_f_, the fluctuation of PNR decreases, and at *T*_f_, the relaxation time of the PNR diverges, which results in the freezing of PNR.^28^ The difference between the *T*_m_ values at 500 Hz and 50 kHz of BCZT19 and BCZT21 is 1.3 and 2.1 K, respectively. The low dispersive behavior around *T*_m_ and close values between *T*_m_ and *T*_f_ denote the weak relaxor nature of the systems. The lower frequency dependency of *T*_m_ can be attributed to the narrow distribution of the size of the PNRs. The relaxation times of PNRs become similar due to their comparable sizes, which causes low dielectric dispersion.^23^

**Figure 5:**
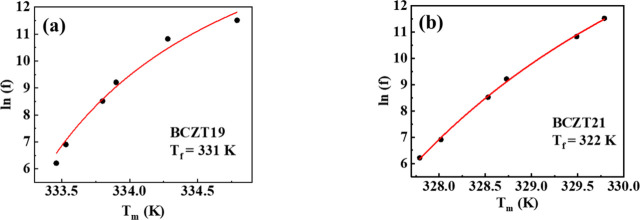
ln­(*f*) vs *T*_m_ graph of (a) BCZT19 and (b) BCZT21 at 1 kHz. The solid curve shows a fitting to the Vogel-Fulcher law.

Figure 6a,b shows the temperature-dependent polarization-electric field (*P*–*E*) hysteresis loops of BCZT19 and BCZT21, respectively. At room temperature, BCZT19 and BCZT21 show saturated P-E loops with maximum polarization values of 16.1 and 16.4 μC/cm^2^, respectively. The temperature-dependent current-electric field (*I*–*E*) loops of the samples are shown in Figure 6c,d. The sharp *I*–*E* loops are observed at room temperature for the compositions. The *P*–*E* loops of the samples become slanted above 363 K, and the peaks in the *I*–*E* loop also decrease significantly above this temperature. The decreasing peak value of the *I*–*E* loop in compositions above 373 K can be explained by the presence of the PNRs above *T*_m_. The contribution of PNRs makes the polarization nonzero, and, as a consequence, the broad peak in the *I*–*E* loop is observed at higher temperatures. Figure 7a shows the variation in maximum polarization (*P*_max_) obtained at an electric field of 5 kV/mm as a function of temperature (*T*) for both compositions. It is worth mentioning here that a gradual decrement in the *P*_max_ is observed with increasing temperature instead of a sharp drop. The *P*_max_ of the materials persists above *T*_m_ also. The positions of *T*_m_ are highlighted in the graphs. Apart from *P*_max_, remnant polarization (*P*_r_) also exists beyond *T*_m_. The variation of *P*_r_ of BCZT19 and BCZT21 with the temperature is shown in Figure 7b. Based on the nature of ferroelectric properties at the higher temperature region, it can be concluded that the existence of polarization above *T*_m_ is associated with the relaxor nature of the compositions. The presence of PNRs and their corresponding polarization above *T*_m_ leads to the nonzero polarization in the temperature range of 333–393 K.

**Figure 6:**
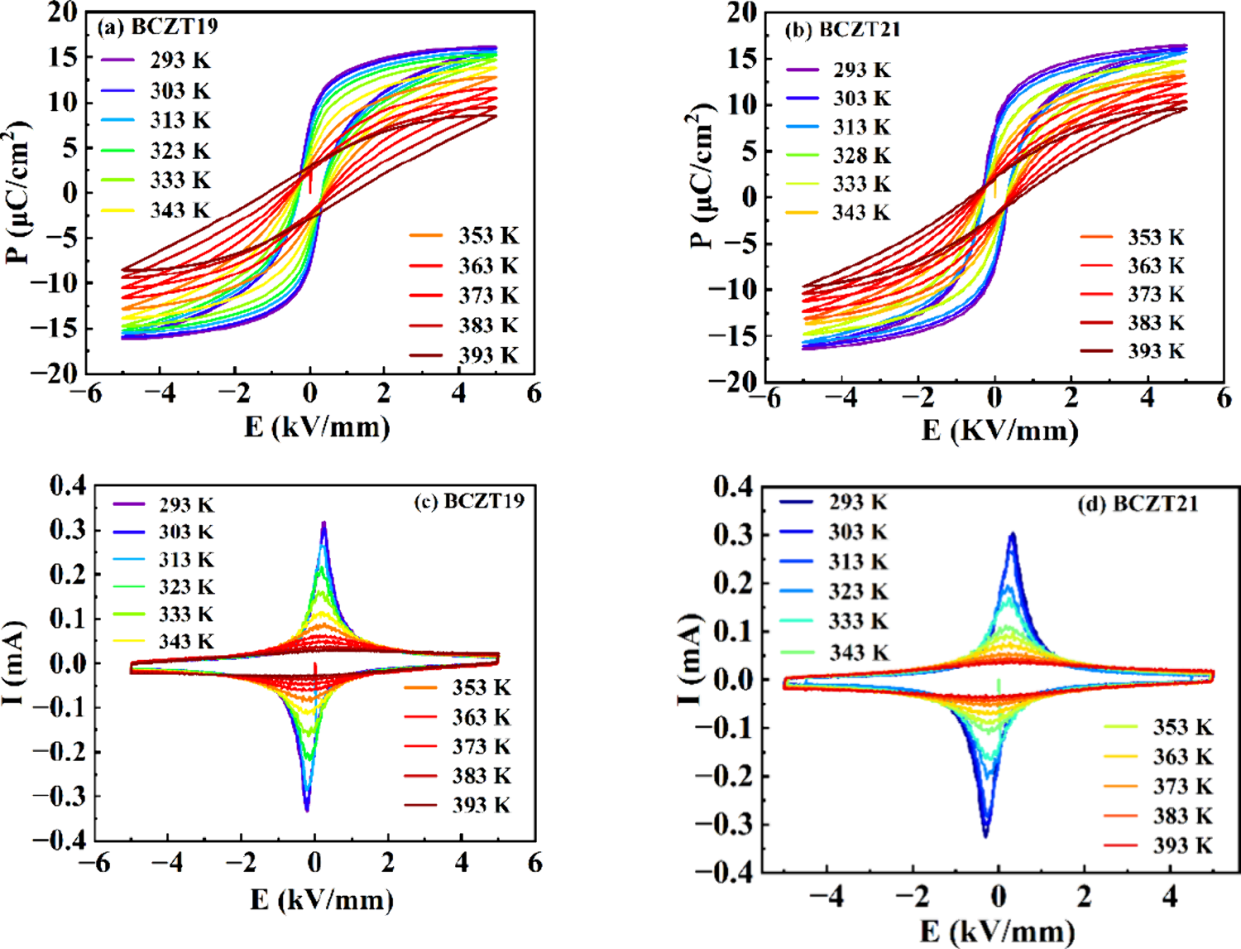
Temperature-dependent (a, b) *P*–*E* and (c, d) *I*–*E* loops of BCZT19 and BCZT21.

**Figure 7:**
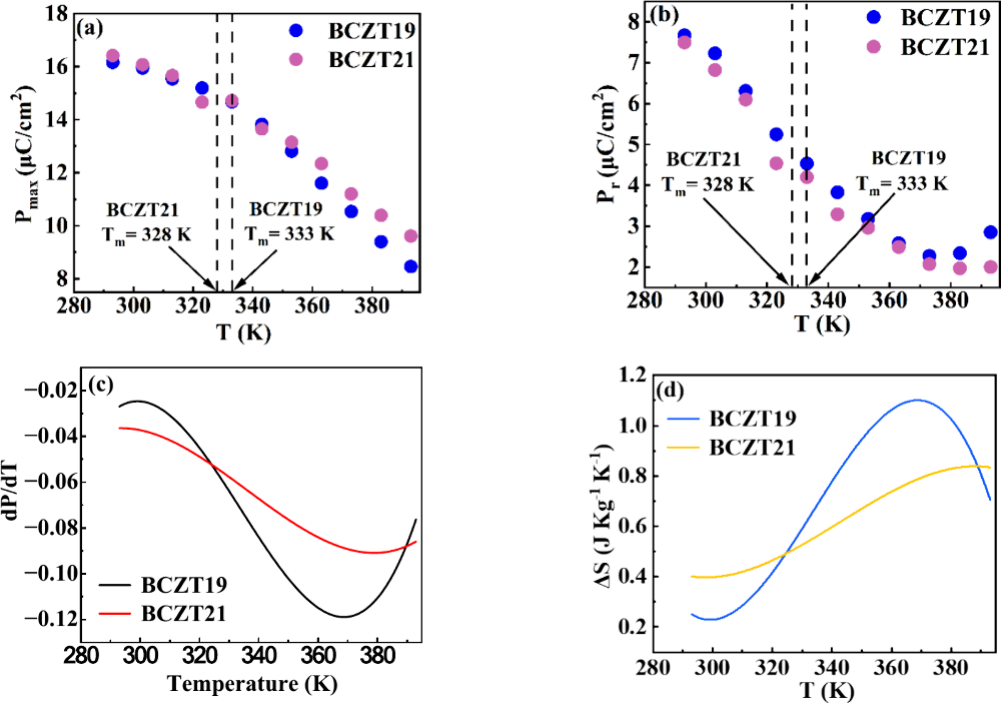
Variation in (a) *P*_max_ and (b) *P*_r_ of BCZT19 and BCZT21 as a function of temperature (*T*), respectively. The positions of *T*_m_ are highlighted by dashed lines. (c) ${\left(\frac{\partial P}{\partial T}\right)}_{E}$ vs *T* plot of BCZT19 and BCZT21. (d) Temperature-dependent Δ*S* of BCZT19 and BCZT21.

The adiabatic temperature change (Δ*T*) and isothermal entropy change (Δ*S*) is calculated based on the Maxwell relation, which is given below:^10^

$${\left(\frac{\partial S}{\partial E}\right)}_{T}={\left(\frac{\partial P}{\partial T}\right)}_{E}$$ 4

The Δ*S* and Δ*T* are calculated according to the following formulas:

$$\Delta S=-\frac{1}{\rho }\int_{E_{1}}^{E_{2}}{\left(\frac{\partial P}{\partial T}\right)}_{E}dE$$ 5

$$\Delta T=-\int_{E_{1}}^{E_{2}}\frac{T}{C\rho }{\left(\frac{\partial P}{\partial T}\right)}_{E}dE$$ 6

Here, ρ and *C* are the density and specific heat of the samples, respectively. *E*_1_ and *E*_2_ are the initial and final applied electric fields, respectively. The ${\left(\frac{\partial P}{\partial T}\right)}_{E}$ is evaluated from the fourth-order polynomial fitting of the *P*–*T* curves (Figure 7c). The values of *C* = 0.4 Jg^1–^K^–1^ are used to evaluate the Δ*S* and Δ*T* for both samples.^9^ The specific heat varies with the composition and temperature. However, their variation is small for the same material system within a limited temperature range. The variation of Δ*S* of BCZT19 and BCZT21 with temperature is shown in Figure 7d. The Δ*S* of the compositions is obtained at 5 kV/mm. The plots of Δ*T* and Δ*T*/Δ*E* of BCZT19 and BCZT21 obtained at 5 kV/mm as a function of temperature are shown in Figure 8a,b, respectively.

**Figure 8:**
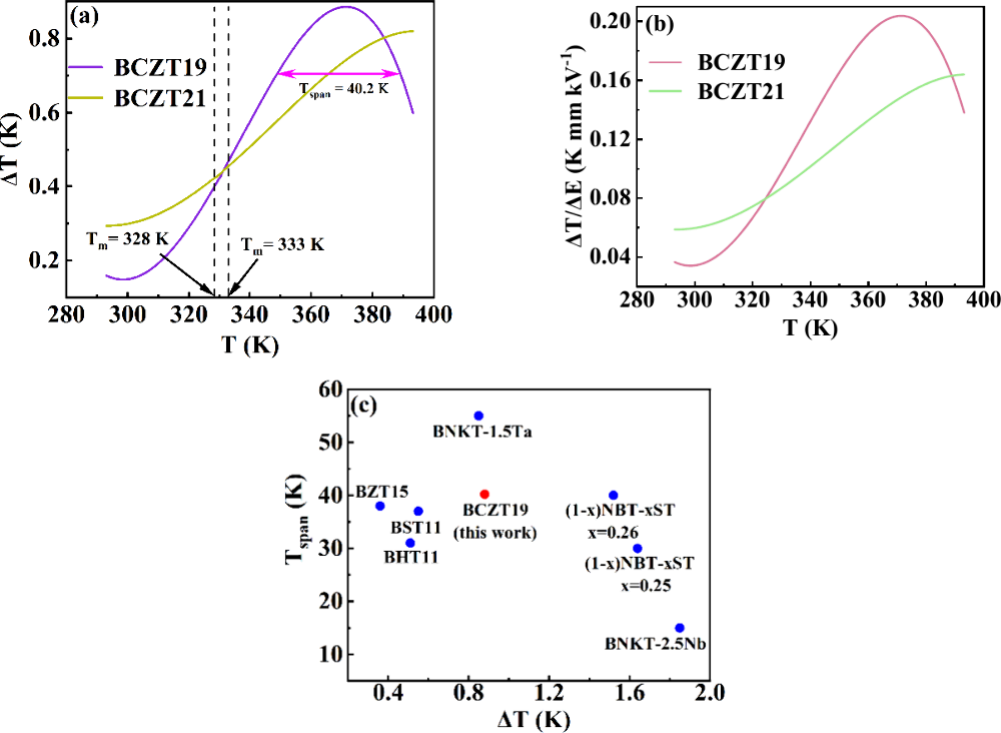
(a) Δ*T* and (b) Δ*T*/Δ*E* as a function of temperature of BCZT19 and BCZT21. The *T*_span_ of BCZT19 is indicated in the Δ*T* – *T* plot. (c) Comparison of the Δ*T* and *T*_span_ values of BCZT19 with other EC materials. The compositions are BZT15 = BaTi_0.85_Zr_0.15_O_3_ [at 20 kV/cm],^16^ BST11 = BaTi_0.89_Sn_0.11_O_3_ [at 20 kV/cm],^16^ BHT11 = BaTi_0.89_Hf_0.11_O_3_ [at 20 kV/cm],^16^ BNKT-2.5Nb= {[Bi_1/2_(Na_0.84_K_0.16_)_1/2_]_0.96_Sr_0.04_}­(Ti_0.975_Nb_0.025_)­O_3_ [at 50 kV/cm],^29^ BNKT-1.5Ta = Bi_1/2_ (Na_0.8_K_0.2_)_1/2_(Ti_0.985_Ta_0.015_)­O_3_ [at 50 kV/cm],^29^ and (1 – *x*)­NBT-xST = (1 – *x*)­(Na_1/2_Bi_1/2_)­TiO_3_ – *x*SrTiO_3_ (for *x* = 0.25 and 0.26) [at 50 kV/cm].^30^

The highest values of Δ*T* (denoted as Δ*T*_max_) and Δ*T*/Δ*E* are 0.88 and 0.20 K/mm kV, respectively, which are observed at ≈371 K for BCZT19. Whereas the highest values of Δ*T* and Δ*T*/Δ*E* (0.81 and 0.16 K/mm kV, respectively) are observed at ≈392 K for BCZT21. The BCZT19 shows higher Δ*T* than BCZT21 due to the higher change in polarization as a function of temperature, as observed from Figure 7c. Here, the Δ*T*_max_ occurs at a much higher temperature than the *T*_m_; the positions of the *T*_m_ of the compositions are highlighted in the figures. This feature can be explained based on the weak relaxor nature and diffuse-type phase transition of the samples. At room temperature, which is below *T*_f_, PNRs are present in the system. With the application of the electric field, a long-range domain order is established in the system. The PNRs are reformed, and the long-range domain order is broken when the temperature is increased. As the temperature is above *T*_f_ and close to *T*_m_, the PNRs are more dynamic, and their ordering becomes random, which enhances the change in entropy. The broad Δ*T*_max_ above *T*_m_ is associated with the contribution of PNRs’ polarization and concentration.

The preservation of the polarization above *T*_m_ is responsible for the existence of the Δ*T* for a wide temperature range. Apart from that, the temperature-dependent ε′ of BCZT19 and BCZT21 shows a diffuse-type phase transition, which denotes that structural phase transitions of these ceramics do not occur around *T*_m_, so there is no sudden drop of *P*_max_ at this temperature. This property of the samples also widens the Δ*T*_max_. Therefore, in the case of the BCZT-based weak relaxor, it can be concluded that the Δ*T*_max_ occurs at a higher temperature than *T*_m_. As anticipated from the diffuseness characteristics of BCZT19 and BCZT21, both samples show a wide *T*_span_. The highest *T*_span_ of 40.2 K (ranging from 388.2 to 348 K) is noticed for BCZT19 (Figure 8a), which is compared with other EC materials and shown in Figure 8c. It is noteworthy to mention here that both high Δ*T* and *T*_span_ parameters are desirable for the practical implementation of the EC material to achieve the thermal stability of the cooling performance. As noted from Figure 8c, BCZT19 is an optimized composition with high Δ*T* and wide *T*_span_ in comparison to other ferroelectrics. The high Δ*T* and wide *T*_span_ of BCZT19 show its potential to provide a stable cooling performance for a broad temperature range.

## Conclusions

In this work, the Pb-free BCZT19 and BCZT21 relaxor ferroelectrics with diffuse-type phase transitions are prepared to obtain a broad *T*_span_ of ΔT. The broad dielectric maxima around *T*_m_ and nonzero *P*_max_ above *T*_m_ of BCZT19 and BCZT21 are attributed to their relaxor nature with diffuse-type phase transition characteristics. The combined contributions of the PNRs and diffuse-type phase transition to the EC effect help to increase Δ*T* and widen the working temperature range of Δ*T*_max_. The BCZT19 shows a Δ*T* of 0.88 K with a *T*_span_ of 40.2 K, which proves the wide working temperature range of the EC effect. The Δ*T*_max_ in BCZT19 and BCZT21 does not occur at either *T*_m_ or *T*_f_, but it occurs much higher than these temperatures. The change in the dipolar entropy associated with PNRs, as well as the diffuse-type phase transition nature, helps to maintain the high Δ*T* as well as the wide *T*_span_ in the compositions. The current findings will help to understand the role of diffusion phase transition on the working temperature range of EC materials and show a pathway to design Pb-free ferroelectrics for EC applications.

## Data Availability

The data that support the findings of this study are available from the corresponding author upon reasonable request.
